# Economic, fiscal, and societal consequences of population aging – looming catastrophe or fake news?

**DOI:** 10.3325/cmj.2020.61.189

**Published:** 2020-04

**Authors:** Jonathan Cylus, Gemma Williams, Charles Normand, Josep Figueras

**Affiliations:** 1 European Observatory on Health Systems and Policies, United Kingdom *j.d.cylus@lse.ac.uk*; 2 Trinity College Dublin, Dublin, Ireland; 3 European Observatory on Health Systems and Policies, Belgium

Populations around the world are getting older. This is happening due to a combination of lower fertility rates and declines in both infant mortality and premature deaths, the latter of which have resulted in longer life spans (1). In high-income countries, there are expectations for large increases in the share of the population above age 80.

While the gains in longevity are roundly celebrated for good reason, there are policy concerns that come with population aging. Many worry whether society is capable of meeting the health care needs of a larger older population. At the same time, there are fears of economic and fiscal consequences with a smaller share of people at traditional working ages (2,3).

However, evidence suggests that many of the common macro-level fears about population aging are exaggerated. This short text draws on research from the European Observatory on Health Systems and Policies program on the Economics of Healthy & Active Aging to consider some of the economic, fiscal, and societal consequences of population aging.

## How will population aging affect health care spending patterns?

In general, the need for health care increases as people age. This contributes to concerns that an aging population will rapidly accelerate health expenditure growth rates. While per person spending does tend to increase with age, there are differences in the magnitude of that increase among European countries. An average 80-year-old in Hungary consumed 16 times more health care in 2015 than the average 20-year-old, but in Cyprus, this difference was only 2.7 times (4).

**Figure 1:**
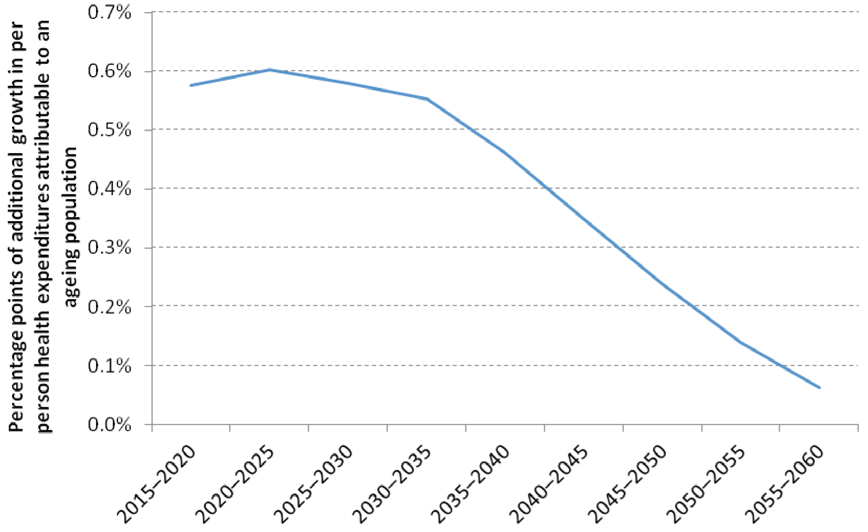
Additional percentage points of growth in per person health expenditure due to population aging, European Union average, 2015–2060. Source (5). Reproduced with permission from the European Observatory on Health Systems and Policies.

However, even though countries spend on average more per person for older people than for young people, population aging is not expected to have a major impact on health spending trends in the future. As has always been the case historically, prices and technological innovation will remain the primary drivers of health spending. Our analysis finds that changes in population age structure will add less than one additional percentage point to average annual per person health care expenditure growth between 2010 and 2060 in the average EU country (Figure 1). Population aging is a slow process that occurs too slowly to become a major health spending driver.

The academic literature suggests alternative explanations for the observed correlation between health spending and calendar age. Researchers find that an individual’s proximity to death or their health status in general can explain the calendar age and health spending relationship (6-8). These factors are naturally important determinants of health spending trends regardless of age. Furthermore, data also suggest that starting at some advanced age, the costs associated with dying decline as people die at progressively older ages (7,9). As a result, as people live longer, it may be that the impact of population aging on health spending growth will be even smaller in the future than we would otherwise expect.

## How will population aging affect the public sector’s ability to fund health care?

Prevailing metrics fuel a misconception that older people are overwhelmingly “dependent” on younger people. The most well-known (and misleading) metric is the old-age support ratio, which assumes that everyone over a certain age (usually age 65) depends on the rest of the adult population for support through their taxes and social contributions to the public sector. Naturally, there are many people over age 65 in formal work and paying taxes, as well as many who are below age 65 who are not. Alternative support ratio metrics aim to define the dependent population more appropriately based on population health or disability status or to explicitly compare consumption and production patterns rather than simply shares of the population above and below an age threshold (10,11). These more refined support ratios suggest that we should not anticipate substantial increases in the share of the population who is dependent and requiring state support in the future. In the United Kingdom, for example, the traditional old-age support ratio is expected to increase from 27% (2005–10) to 41% (2045–50). However, the share of the adult population who is disabled is projected to remain constant over time at around 10%.

It is important to move even further beyond old-age support ratios to have a clearer sense of how population aging affects public sector revenues. In European countries, the main sources of public sector finances are income taxes, goods and services taxes, property taxes, and social contributions (12). Each of these is likely to be affected by changing age-demographics.

**Figure 2:**
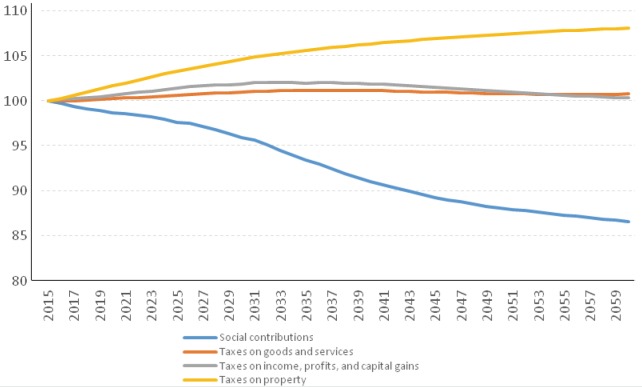
Changes in per person tax and social contributions as the Japanese population ages (index 2015 = 100), 2015-2060. Source (13). Reproduced with permission from the European Observatory on Health Systems and Policies.

Simulations allow us to quantify precisely how population aging can affect public sector revenues. Population data from Japan, a country with a large older population that is increasingly entering retirement, suggest that relying on labor markets (ie, social contributions) to raise public sector revenues and pay for health and other services will lead to fewer public revenues per person over time (13). Other forms of taxation, such as taxes on goods and services or income taxes, appear to be impervious to changing age-demographics (Figure 2).

This can have important implications for health financing, especially in settings that rely heavily on labor market contributions for funding. Possible policy options to address this challenge include increasing labor market participation at older ages; increasing social contribution rates; and diversifying the mix of revenue sources that are used to finance health. However, in health systems that depend on social contributions there appears to be no simple solution to keep stable revenues. Diversifying funding toward revenue sources that are less affected by population aging may help somewhat. However, a combination of re-prioritization of public sector budgets in favor of health and increased rates of taxation will likely be needed to maintain adequate, stable revenues for the health sector (13).

## How will population aging affect formal and informal work?

While population aging will in all likelihood alter economic behaviors that have consequences for public sector and health sector revenues, as discussed above, there remains great scope for older people to contribute to society and to the economy, especially if they can remain healthy and active. Many people remain in paid work at older ages (14). Labor productivity may change to varying degrees for different types of work as people age; for example, jobs that are not so physically demanding may see productivity increase alongside more years of experience. In fact, even without distinguishing between jobs that require considerable physical exertion and those that do not, empirical evidence suggests that on average many people have considerable health capacity to continue to work at older ages (15,16).

**Figure 3:**
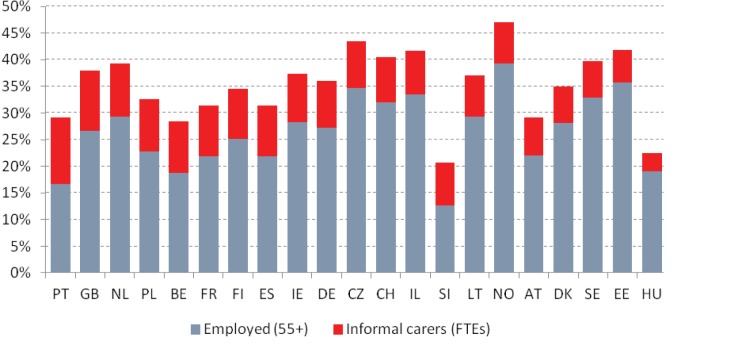
Employment rates including informal caregiving (adjusted for full time equivalency) among people older than 55, selected European countries. Source (3). Reproduced with permission from the European Observatory on Health Systems and Policies.

A key question therefore is whether many older people are already contributing to the economy and society in unrecognized ways. For example, informal caregiving is commonly done by older people. If informal carers were accounted for in the same way as paid workers in national employment statistics, there would be a considerable increase in the employment rates for older people, which may to some extent alter ageist perceptions that older people do not contribute sufficiently to society. In Portugal, accounting for informal carers as any other job would result in an almost 13 percentage point increase in employment rates among those over 55 (Figure 3). Put another way, one study from Spain estimates the economic value of informal care being in excess of 2% of GDP (17).

## Conclusion: how should decision-makers respond to population aging?

This text finds that alarmist viewpoints suggesting that population aging will lead to untenable fiscal pressures and that it will erode the welfare state are overblown, if not simply false. Particularly from a health system’s perspective, population aging will not lead to exorbitant expenditure growth, though it may require a re-think in some countries about the most appropriate and sustainable funding mechanisms and how best to organize services. At the same time, a careful look at the data confirms that older people – particularly those who are healthy and can remain active – provide a number of readily quantifiable benefits that are often overlooked by common metrics and national statistics.

Even if aging does not pose an existential threat, there remains great scope for policy interventions to ensure population aging is a boon, rather than bust. Without a doubt, policies that support the health and functional capacity of older people are essential, both because of the economic gains associated with good health through lower care costs and greater work potential, as well as because of the intrinsic value of good health. Political debates on whether and how to support the health and activity of older people should carefully consider the evidence on the probable costs as well as the benefits that come with healthy and active population aging.
